# Ancient DNA studies: new perspectives on old samples

**DOI:** 10.1186/1297-9686-44-21

**Published:** 2012-07-06

**Authors:** Ermanno Rizzi, Martina Lari, Elena Gigli, Gianluca De Bellis, David Caramelli

**Affiliations:** 1 Institute for Biomedical Technologies, National Research Council, Via F.lli Cervi 93, Segrate, Milan, 20090, Italy; 2 Department of Evolutionary Biology, Laboratory of Anthropology, Molecular Anthropology/Paleogenetics Unit, University of Florence, Via del Proconsolo 12, Florence, 50122, Italy

## Abstract

In spite of past controversies, the field of ancient DNA is now a reliable research area due to recent methodological improvements. A series of recent large-scale studies have revealed the true potential of ancient DNA samples to study the processes of evolution and to test models and assumptions commonly used to reconstruct patterns of evolution and to analyze population genetics and palaeoecological changes. Recent advances in DNA technologies, such as next-generation sequencing make it possible to recover DNA information from archaeological and paleontological remains allowing us to go back in time and study the genetic relationships between extinct organisms and their contemporary relatives. With the next-generation sequencing methodologies, DNA sequences can be retrieved even from samples (for example human remains) for which the technical pitfalls of classical methodologies required stringent criteria to guaranty the reliability of the results. In this paper, we review the methodologies applied to ancient DNA analysis and the perspectives that next-generation sequencing applications provide in this field.

## Review

### Twenty-eight years of ancient DNA

The field of ancient DNA studies began twenty-eight years ago with the extraction and sequencing of DNA material from the quagga, a South African equid (*Equus quagga quagga*) that went extinct in the 19^th^ century [1] and from an Egyptian mummy [2]. These studies used bacterial cloning to amplify small DNA sequences (retrieved from skin fragments of these specimens) and showed that the origin of the extracted genetic material was mainly microbial or fungal. In general, endogenous DNA was composed of very low concentrations of short and damaged fragments of multi-copy loci, such as mitochondrial DNA (mtDNA).

A few years later, with the development of PCR (polymerase chain reaction) it became possible to routinely amplify and study surviving ancient DNA molecules even if only in a single copy, resulting in a rapid increase and diversification of ancient DNA research [3-5]. However, due to the enormous power of PCR to amplify even a few copies of DNA sequences, modern DNA contamination has become a crucial problem. For this reason, many of the most extravagant reports on ancient DNA, including claims of DNA sequences surviving for millions of years in plants [6-8] and dinosaur bones [9], have been disputed and actually disregarded. Studies on ancient DNA need to deal with technical problems that are specific to this field. The first difficulty is the production of sufficient quantities of authentic DNA sequences to make a study conclusive. This difficulty is a consequence of post-mortem DNA degradation processes, which can cause miscoding lesions, potentially leading to sequence errors, or physical destruction of the DNA molecule, thus increasing the risk for preferential amplification of exogenous contaminant sequences (Table 1). To deal with this issue, researchers have outlined a series of guidelines to ensure the quality of ancient DNA data and the reliability of consequent conclusions [5]. Over the years, these guidelines have gradually evolved into a more detailed and extensive list of requirements, resulting in the nine “gold criteria” outlined by Cooper and Poinar in “Ancient DNA: do it right or not at all” [10], one of the most cited papers in the field (Additional file 1). In particular, the authors have suggested that, in the absence of full compliance with all nine criteria, the reliability and authenticity of results remain uncertain.

**Table 1:** Ancient DNA damage

**Damage**	**Type of process**	**Effects on DNA molecule**	**Possible solutions in aDNA classical sequencing methodologies**
Oxidative damage	Formation of strand breaks (single-stranded nicks)	Cleavage of the phosphodiester backbone	PCR of overlapping fragments of short length
		Depurination resulting in a baseless site	Multiple independent PCR Cloning and sequencing of several clones
		Breakage of the sugar backbone through b-elimination	Uracil-N-glycolase treatment
		Results in lesions blocking the polymerase enzyme, and promoting chimeric sequences through ‘jumping’ PCR	Blocking primers Single primer extension or Spex
Degradation by microorganisms’ nucleases in the post mortem cell	Strand breaks	Short fragment length	PCR of overlapping fragments of short length
DNA crosslinks	Inter-strand crosslinks by alkylation	May prevent the amplification of endogenous template molecules	PTB (N-phenylacyl thiazolium bromide)
	Intermolecular crosslinks by Maillard reaction	Increases the risk of contamination	
Hydrolysis damage	Results in miscoding lesions, for example, deamination of cytosine and adenine to uracil and hypoxathine, respectively	Results in the incorporation of erroneous bases during amplification and change of coding	Multiple independent PCR Cloning and sequencing of several clones UNG treatment

* UNG* : Uracil-N-glycosylase; *Spex*: Single Primer Extension; *PTB*: N-phenylacyl thiazolium bromide.

In the last few years, with the advent of new sequencing technologies, the field of ancient DNA is experiencing a new era wherein what was once impossible is now possible, as for example drafting genomes of extinct organisms such as *Homo neanderthalensis*[11], or distinguishing endogenous from contaminant DNA in archaic *Homo sapiens* specimens [12]. In this report, we review the history of studies on ancient DNA from a methodological point of view, ranging from the most significant ones performed with the so-called “classical methodology” consisting in PCR amplification, cloning and traditional Sanger sequencing, to the more recent ones performed by next-generation sequencing technologies (NGS), which are promising to revolutionize the field of ancient DNA.

### What was achieved with the classical methodology?

Starting from the first pioneering studies in the 1980s, classical methods to recover and analyze DNA from ancient specimens have been developed, and continuously improved, to overcome the two main technical limitations characterizing this field, e.g. the poor preservation of endogenous DNA and the presence of contaminant exogenous DNA.

Basically, the traditional methodology consists in three fundamental steps:

1. PCR amplification of several short and overlapping target fragments (60–200 bp long) to recover larger regions.

2. Production and sequencing of several clones for each amplified fragment.

3. Alignment and comparison of sequences from different clones and different overlapping fragments to reconstruct the final consensus sequence of the entire region of interest.

Using this protocol, Krings et al. [13] have reported in 1997, the first reconstruction of a DNA sequence from an extinct hominin, *Homo sapiens neanderthalensis*. This consisted in a small fragment of mitochondrial DNA (mtDNA) [13] that was subsequently enriched by other mtDNA sequences from the same individual [14]. A few years later, these results were corroborated by additional mtDNA sequences from other Neanderthal specimens distributed all over Europe [15-19].

Until recently, almost all the genetic studies performed on ancient specimens targeted mtDNA regions. Cells contain 100 to 10 000 copies of mtDNA that constitute a primary source of DNA in ancient specimens. In addition, the mitochondrial genome being maternally inherited and not subjected to recombination, mtDNA mutations are transmitted clonally across generations and can be used to trace maternal lineages. Because of these characteristics, mtDNA has been successfully used in several studies to reconstruct the phylogenetic relationships between extant and extinct species, such as Australian marsupial wolves [5,20], New Zealand moas [21,22], American ground sloths [23] endemic Hawaiian goose [24], cave bears [25], Balearic Islands cave goats [26], giant lemurs [27] and Caspian tigers [28].

The preservation of many individuals, all originating from a single locality, either as museum specimens previously collected by naturalists, or specimens retrieved by archaeologists at a single site, provides an opportunity to track changes in a population over time. The first example of this approach is a study on three populations of kangaroo rats in California, which were collected by zoologists in the first half of the last century. Comparison of modern populations from the same area has shown that the mitochondrial lineages are spatially stable, a situation typical of undisturbed habitats [29]. A more recent study conducted on mice sampled in Chicago (USA) has demonstrated that, in this population, mitochondrial lineages have been replaced over the last 150 years, probably due to human influence [30]. Similar studies have traced the population history of many other species including rabbits [31], pocket gophers [32], black-footed ferrets [33], sea otters [34], grizzlies [35], red squirrels [36], canids [37], penguins [38], artic fox [39], Chatham Island taikos [40], woolly mammoths [41], bears [42], equids [1,43], Tasman booby [44] and quails [45]. Ramakrishnan and Hadly [46] have reviewed these studies and reanalyzed some datasets. They concluded that genetic samples collected from populations over large temporal and geographical scales, when analyzed using complex models and the serial coalescent approach, are critical to understand past population dynamics and provide important material to reconstruct evolutionary processes.

An interesting study on late Pleistocene brown bears has radically revolutionized the current knowledge about bear population dynamics in Alaska [47]. Whereas present-day brown bear mitochondrial lineages are neatly distributed in different geographical areas, this study has shown that, about 35 000 years ago, the same mitochondrial lineages all coexisted in a single area. This result has crucial implications for conservation genetics. Mitochondrial lineages that are spatially separated today are often considered to have separated a long time ago and to be carried by “subspecies” adapted to different environments. As a consequence, it is often suggested that they should be kept separated and not be allowed to mix in captivity or during the restocking of wild species. The data on ancient DNA sequences from brown bear prove that contemporary samples may not reproduce long-term patterns. In the future, it can be predicted that direct testing of the phylogeographic patterns of additional species will clarify whether or not these results reflect recent effects of random genetic drift within small populations or, whether they represent long-term separation of populations.

Studies on ancient DNA have also been used to investigate the demographic history of human populations although such studies may suffer from contamination problems. One of the earliest publications in this field is a population genetics study on the Etruscans, a prehistoric Italian population [48]. Other studies have examined the origins of Andaman Islanders [49], the genetic variations in prehistoric Iberians and Sardinians [50,51], and the genealogical discontinuities among Etruscans, Medieval and contemporary Tuscans [52]. Particular attention has also been paid to the investigation of the genetic relationship between local hunter-gatherers and the first farmers during the Palaeolithic-Neolithic transition in Europe [53-55]. Although based on reduced sample sets, these studies revealed a genetic discontinuity between these populations.

Other studies on ancient DNA have aimed at tracing the genetic history of domestication. The most extensively studied species are cattle and pigs. The limited geographical range of sheep and goat wild ancestors indicates that the domestication of these species probably occurred in Anatolia and in the Fertile Crescent. On the contrary, the wild progenitors of cattle and pigs are distributed on a wider area indicating that their domestication scenario may be more complex with possible genetic contributions from local wild stocks.

The first report on ancient bovine DNA concerns the mtDNA sequences of the extinct wild oxen (*Bos primigenius*) from Britain [56,57]. Phylogenetic analysis of *Bos primigenius* ancient sequences has shown that these animals are clearly distinct from modern cattle. For this reason, they have been assigned to a different clade, named “P”, in contrast to the “T” clade corresponding to modern domestic cattle. Even if based on only a few sequences from a single mtDNA region, the data point to a Near-Eastern origin of European cattle. More recent genetic studies on aurochsen and ancient domestic cattle from different locations have suggested that the process of cattle domestication may be more complex than previously thought [58-62]. When analyzing mtDNA from Pleistocene Italian aurochsen, Beja-Pereira et al. [59] and Mona et al. [62], have found that the vast majority of the Italian sequences fall within the range of variation observed in modern cattle (“T” clade) and are genetically distinct from the “P” clade of the aurochsen from the British Isles [62]. In 2007, Edwards et al. have reported that only “P” type sequences are present in ancient aurochsen from North and Central Europe [61], while nearly all the sequences of the first domestic cattle of the Neolithic and Bronze Ages belong to the “T” clade [58,60]. These data represent good examples of how studies on ancient DNA can contribute to shed light on complex scenarios characterizing cattle domestication and diffusion processes. Indeed, populations domesticated in the Near East and introduced into Europe during the Neolithic diffusion might have intermixed in Southern European regions with local wild animals carrying mtDNA of the “T” type. In addition, a greater genetic variability has been observed in aurochsen from South Europe than in those from North and Central Europe. Previously unknown non-“T” mtDNA sequences have been discovered in some modern local Italian breeds [63-65]. As a consequence, European breeds could represent a more diverse and important genetic resource than previously recognized, especially in Southern regions.

Two important papers by Larson et al. [66,67] based on ancient mtDNA sequences of both wild and domestic pigs from different times and locations, have provided insights into pig domestication processes and Neolithic expansion in Europe, as well as in East Asia and Oceania. By analyzing several ancient pig specimens collected across West Eurasia, the authors have shown that Near Eastern pigs have been introduced by humans into Europe and that they may have traveled along at least two distinct routes (the Danubian corridor and a southern maritime route that ran through the North shore of the Mediterranean) [67]. Then, European wild boars were domesticated and rapidly became the predominant lineage within the European domestic swine. In the light of these results, the process of pig domestication in Europe appears fundamentally different from that in the Near East. In Europe, it may not have been a truly independent event, but rather a direct consequence of the introduction of Near Eastern domestic pigs by early farmers. Other similar studies have investigated the domestication process of chicken and horse [68,69].

### Improvements of the classical methodology

Since, even when preserved in ideal conditions, DNA degrades over time, the process of amplification, cloning and sequencing is usually constrained by the short length of the DNA fragments recovered and by the small amount of template DNA in the sample. Although longer sequences are more informative, in practice, with the classical methodology, only overlapping shorter sequences are available that must then be meticulously assembled together. For this reason, until 2005 it was not possible to obtain DNA sequences longer than 1000 base pairs (bp), even from widely studied Pleistocene mammalian species such as mammoths, ground sloths and cave bears. With the breakthrough in ancient DNA sequencing due to the innovative multiplexing strategy of Krause et al. [70] to reconstruct long DNA sequences from several shorter fragments, the complete sequence of the 16 770 bp mitochondrial genome of the Pleistocene woolly mammoth (*Mammuthus primigenius*) was reported, starting from only about 200 mg of bone. Basically, the multiplexing strategy consists of a two-stage PCR: (1) first, multiple primer pairs are used to target subsequences within the complete DNA sequence and (2) the amplification product is divided in as many aliquots as the number of primer pairs used in the multiplex PCR and each aliquot serves as template for individual secondary PCR that separately amplify each target. For the woolly mammoth mtDNA, 46 such primer pairs were chosen to amplify overlapping DNA sequences covering the entire mtDNA genome. Then, primer pairs were divided into two sets, each one comprising primers amplifying alternate non-contiguous fragments. Multiplex PCR was then performed with only as much ancient DNA template as would be normally used for short target sequencing. Even if the multiplexing approach does not save time (many secondary PCR are still required), it certainly saves DNA material, since it can use hundreds of primer pairs in the same tube, thus overcoming typical limitations in sample amount.

Modifications have been made to this classical methodology in order to explore the molecular nature of the DNA damages occurring in such specimens. One common feature of ancient DNA samples is the presence of miscoding lesions that cause the incorporation of incorrect nucleotides during DNA amplification. In 2001, Hofreiter et al. showed for the first time that most of these damage-derived errors are caused by hydrolytic deamination of cytosine into uracil leading to apparent C → T or G → A substitutions in DNA templates sequenced after PCR amplification [71]. To reduce the number of such misincorporations, assays involving pre-treatment of ancient DNA samples with uracil N-glycosylase have been conducted to remove uracil residues from the DNA sequence and leave abasic sites that prevent replication by the Taq polymerase during PCR. However, the use of uracil N-glycosylase has not been widely adopted in studies with ancient DNA, because the number of template molecules in such samples is often so small that the enzyme may destroy all the amplifiable templates. In 2007, Cooper et al. reported another approach, i.e. Single Primer Extension (SPEX), which can provide detailed information about post-mortem base modifications in ancient DNA [72]. Unlike PCR, SPEX is an amplification methodology that uses single biotinylated primers to specifically target only one strand of the ancient DNA template at a locus of interest without imposing any predefined target length. With SPEX, primer extension continues until it is stopped by the end of the template due to fragmentation or by a polymerase-blocking lesion. After the primer extension step, all non-biotinylated DNA templates are removed by washes in the presence of streptavidin beads and the remaining first-generation copies of the ancient DNA target are then ligated to a polyC-tail and amplified by nested PCR. Comparison of data obtained with SPEX amplification and standard procedures on the same samples showed that SPEX can produce sequence data of unprecedented accuracy from ancient DNA, without introducing additional exogenous sequence artifacts. Cooper et al. [72] also suggested that the C → U base modifications may originate from the damage-derived miscoding lesions present at significant levels in samples of ancient DNA. Despite its potential interest, after this initial report, SPEX has not been widely applied mainly because of its intrinsic laborious protocol compared to other more innovative procedures (see “Next Generation Sequencing methodology” section).

Another recent implementation in the classical methodology is the use of blocking oligonucleotides that preferentially bind modern contaminant DNA and prevent its amplification, in combination with standard primers specific for the target of interest. A blocking oligonucleotide consists in modifying its 3’ end with a C3 spacer to prevent the TaqDNA polymerase from extending it without significantly changing its annealing properties to the target DNA. The C3 spacer is a standard primer modification available from most suppliers of custom oligonucleotides and consists in a short three-carbon spacer arm [73]. This novel PCR method has been tested by Gigli et al. on four Neanderthal samples with different contamination levels and taphonomic conditions [74]. Usually, Neanderthal skeletal remains are contaminated with modern human DNA derived from the handling and the washing of the specimens during excavation. Although Neanderthal and modern-human haplotypes differ, allowing the design of specific primer pairs for most of the mtDNA hypervariable region 1 (HVR1), the human contaminants can often outnumber the endogenous DNA, thus preventing a successful retrieval of Neanderthal sequences. However, including blocking oligonucleotides, specifically designed to preferentially bind modern-human specific sequences from contaminant sources, has made it possible to significantly increase the yield of Neanderthal DNA sequences in all four samples (from 25.23 % up to 90.18 %) by inhibiting the hybridization of the standard primers.

### Nuclear ancient DNA

Even if a sample of ancient DNA, typically contains 1000 times more copies of mtDNA than nuclear DNA, the number of mtDNA copies, sufficiently well preserved to be analyzed, is very low. In fact, nuclear DNA seems less prone to degradation and damage over time, so the chance of recovering longer intact strands is actually greater for nuclear DNA than for mtDNA. In addition, DNA damages are less frequent in nuclear DNA than in mtDNA, possibly because nuclear DNA is better protected by proteins.

From a quantitative point of view, DNA sequences that occur in many hundreds of copies per cell, such as DNA from mitochondria or chloroplasts in plants, are more easily retrieved from ancient specimens than are nuclear DNA sequences that occur only once per haploid genome. Therefore, phylogenies are usually reconstituted with information from only a few genetic loci, which limits phylogeny reconstruction of species that have diverged either recently or so rapidly that different parts of their genome have different phylogenies. However, this limitation can be overcome in some cases. For example, it has been possible to determine nuclear DNA sequences from several Pleistocene animals [75,76], from plants preserved in dry environments [77] and from museum specimens [78,79]. An interesting study on ancient cattle samples has investigated cattle domestication based on paternally transmitted Y chromosome-specific single nucleotide polymorphisms (SNP) [80,81]. In modern European cattle, one SNP in intron 19 of the Y-chromosomal gene *UTY* and one 2-bp insertion–deletion polymorphism in the *ZFY* intron 5, distinguish two haplogroups: Y1, primarily found in Northern Europe, and Y2, mainly distributed in Southern Europe [80]. Detection of the Y1 haplogroup in ancient cattle and aurochs from Northern and Central Europe has been initially interpreted as proof of a hybridization event having occurred in Europe between domestic cows and aurochs bulls [80]. However, subsequent studies on different sample sets have failed to find the same pattern, and shown a great fluctuation of allele frequencies over time [81,82]. These results suggest that the present-day frequencies of Y1 and Y2 haplogroups probably depend more on recent demographic events than on events having occurred in the first stages of cattle domestication. Even if the analysis of Y chromosome lineages is potentially useful to understand the evolutionary history of cattle, there is still no patrilinear marker capable of tagging a possible introgression of European aurochs into domestic cattle.

Having access to nuclear ancient DNA might enable scientists to investigate ancient animal and human phenotypes, such as skin color and behavioral traits. Of all the important questions on Neanderthals, that of whether they were able to speak and, if so, how well, is a key issue. To address this, Krause et al. [83] have analyzed the *FOXP2* gene, a gene intimately connected with the ability to speak in humans. They have applied the classical multiplexing approach, to characterize two SNP in the *FOXP2* gene and to check a number of modern human DNA contamination control positions on autosomes, sex chromosomes and mtDNA. They have found that Neanderthal and modern humans share the same polymorphisms at the *FOXP2* locus, which differ from those found in all other mammals, suggesting that *Homo sapiens neanderthalensis* most probably had the ability to speak like modern humans. In addition, the reconstructed Neanderthal Y chromosome sequence falls outside the range of variation observed in modern humans, indicating that the *FOXP2* sequences obtained are authentic and confirming that the Neanderthal paternal contribution to the human nuclear genome is low.

During the same period, Lalueza-Fox and colleagues have amplified and sequenced a fragment of the *melanocortin 1 receptor* (*MC1R*) gene from two Neanderthal remains [84]. The *MC1R* gene regulates pigmentation in humans and other mammals. In particular, *MC1R* variants with a reduced function are associated with pale skin color and red hair in humans of European origin. Lalueza-Fox et al. have shown that both Neanderthal specimens carry a mutation in the *MC1R* gene that is completely absent in the 3700 samples analyzed from modern humans. Furthermore, functional analyses have revealed that this variant reduces MC1R activity to a level that alters hair and skin pigmentation in humans. These results suggest that Neanderthals varied in pigmentation levels, potentially on the same scale as observed in modern humans, and, in addition, that defective MC1R variants have evolved independently in modern humans and in Neanderthals.

An interesting study by Ludwig et al. [85] based on ancient samples representing different periods between the Late Pleistocene and the Middle Ages and targeting nuclear genes responsible for coat pigmentation, has shed light on the timing and place of horse domestication. This study has shown that the rapid increase in number of coat color patterns is a result of domestication and the great variation in coloration of modern domestic horses is a result of selective breeding by ancient farmers.

### The problem of contamination in the classical methodology

Contamination is a serious problem in studies with ancient DNA, even more so when dealing with human ancient DNA than with animal or plant ancient DNA. Early results from human remains collected in hot climate regions, such as Egypt [2] and Florida [86], are now considered as probably coming from modern human contaminations. For example, a 3.4 kb long nuclear DNA sequence reported as amplified from an Egyptian mummy is an unusually long sequence for DNA extracted from ancient remains. In addition, it is well known that high temperatures such as those occurring in Egypt do not help DNA preservation [87]. Several papers have reported that, despite the use of rigorous protocols [10,88], modern human DNA contamination is prevalent in amplification products from DNA extracts of ancient specimens [13,88-91]. In fact, it has been shown that even with extensive UV and bleach treatments, it is impossible to completely remove modern human DNA from ancient bones and teeth [92]. This is probably due to the porosity of bone and tooth dentine, which are the main entry routes for DNA coming from sweat, skin fragments and exhaled cells. However, when teeth are well preserved and directly removed from the jaw or maxilla, they appear less prone to contamination than bone fragments. Hair is also a more reliable source for studies on human ancient DNA, since it seems to be less susceptible to contamination than bone and teeth, even if its presence is less common in ancient specimens than skeletal remains [93]. In a different study, Salamon et al. [94] has shown that relatively well preserved DNA can be present within clusters of inter-grown bone crystals that are resistant to sodium hypochlorite (NaOCl) disaggregation, a strong oxidant. These authors obtained reproducible authentic DNA sequences from bone crystal aggregates of both modern and ancient animal bones, including humans. Moreover, they claim that the NaOCl treatment minimizes the risk of modern DNA contamination. Indeed, applying this treatment on fossil bones, they have demonstrated the presence of a privileged niche containing DNA in a better state of preservation than DNA extracted from the total bone. Thus, this approach could significantly increase the chances of obtaining authentic ancient DNA sequences, especially from human bones.

For all these reasons, when analyzing ancient human DNA samples with the classical methodology, it is essential to collect skeletal material that has been manipulated only with disposable gloves and face-masks during excavation (Additional file 2), and to select archaeological remains for which the taphonomic history is well-known.

Many excavated archaeological specimens appear to contain DNA from multiple individuals [95,96], which raises the issue of how to authenticate ancient human DNA sequences, when they do not differ from DNA from potential contaminants (modern Human) such as Neanderthals [13] or distinct modern human groups like the Andaman Islanders [49]. A good example is the analysis of Italian Cro-Magnon specimens [97], which have been submitted to comprehensive authentication protocols [10,88]. However, since the sequences obtained are indistinguishable from those of modern European humans, sample contamination must remain the null hypothesis. Another serious issue is the presence of modern human DNA in samples that were not expected to contain any [13,15,88,94,98-100]. This has been carefully investigated in two studies on Neolithic and Upper Palaeolithic samples, respectively by Sampietro et al. [50] and Caramelli et al. [101]. Sampietro et al. [50] have characterized the mtDNA hypervariable region 1 (HVR1) in 23 Neolithic remains excavated from Granollers (Barcelona, Spain). They compared the sequences obtained with those from the DNA of the six persons involved in the excavation, the washing and subsequent anthropological and genetic studies of the specimens. They found that 17.13 % of the 572 cloned sequences generated from ancient DNA extracted from the teeth of the Neolithic specimens could be unambiguously identified as contaminants derived from the persons involved in the project. Moreover, when checking the cloned contaminant sequences, they observed that the level of damage in the contaminant DNA molecules increased with time and demonstrated that the damage rate of the older contaminant and the endogenous DNA sequences were indistinguishable. Therefore, the commonly used argument that miscoding lesions observed in cloned ancient DNA sequences can be used to support data authenticity is misleading in scenarios where the presence of old contaminant sequences is possible. For this reason, these authors claim that genetic typing of people involved in the manipulation of ancient human specimens is critical to ensure the accuracy and authenticity of findings. In 2008, Caramelli et al. performed the genetic characterization of a Cro-Magnoid individual, Paglicci, from layer 23, whose taphonomic history was very well known [101]. Consequently, they were able to monitor all the possible contaminations from persons having manipulated the sample, and demonstrated that the sequence obtained from the Paglicci bones differed from those of all the people that had been in contact with the bones. In addition, since the sequence obtained for the Paglicci mtDNA HVR1 is still common in European humans and since no HVR 1 sequence similar to that of Neanderthals is present in modern Europeans, they conclude that Neanderthals probably did not contribute to the modern human mitochondrial gene pool. In 2010, Krause et al. confirmed this conclusion in a study involving more innovative techniques that will be described in the next sections [12].

### Next Generation Sequencing (NGS) methodologies

As described in the above section, in the pre-NGS era, PCR and Sanger sequencing were the main tools available to analyze ancient DNA samples. The development of miniaturized gel electrophoresis (capillary electrophoresis) and the automation of reactions, gel loading and signal detection allowed the Sanger methodology to become the gold standard for DNA sequencing. Despite these features, Sanger sequencing has a low throughput and consequently is expensive for large-scale sequencing. Moreover, library preparation and amplification, and colony picking have a low efficiency and are time-consuming steps. With ancient DNA, these drawbacks are even more critical and the development of NGS has opened up whole new possibilities and extended the field of applications [102].

### NGS methodologies: the background

**Figure 1:**
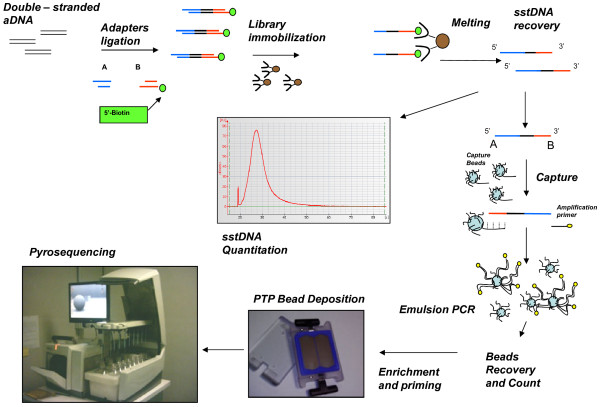
**454/Roche NGS procedure.** Double-stranded ancient DNA is converted in single-stranded DNA library through the ligation of specific 454 “adapters” (A and B); the emulsion PCR amplifies the library molecules on “capture beads” that are then enriched and loaded onto PicoTiterPlate (PTP) for the pyrosequencing reaction.

**Figure 2:**
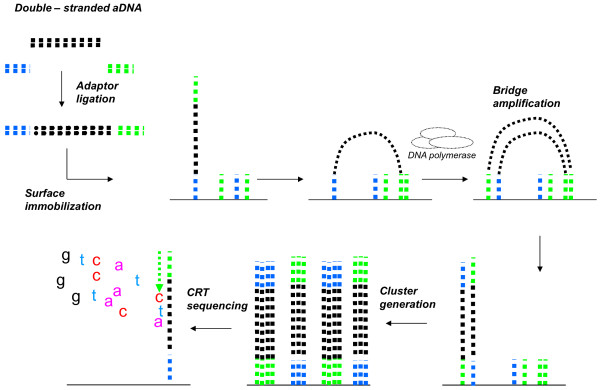
**Illumina sample procedure.** Double-stranded ancient DNA is converted into an Illumina library and is then amplified by “bridge amplification” onto the surface of the “flowcell”; amplified molecules are sequenced by the cycle reversible termination (CRT) methodology.

The new generation of NGS sequencers has made it possible to increase the number of bases sequenced per run with a concomitant decrease in sequencing costs, due to technological improvements. The most important NGS platforms used in the field of ancient DNA analyses are the 454/Roche FLX and the Illumina Genome Analyzer [102-104]. Both technologies share the same rationale for the production of sequences (reads), but differ in the amplification procedure and sequencing chemistry resulting in different throughputs. In spite of the high sensitivity and productivity of NGS sequencers, their signal detection system is not sensitive enough to measure the sequencing signal originating from a single molecule. Detection systems, such as the CCD camera for both 454/Roche and Illumina, can identify a signal only if it is generated by millions of DNA molecules, thus amplification of the sequencing library is necessary. The three key steps for generating reads are: (1) library preparation, (2) library amplification and (3) sequencing. More details about these steps are described and illustrated in Additional file 3 [see Additional file 3] and in Figures 1 and 2.

#### Library preparation

For both the 454/Roche and Illumina platforms, it is necessary to prepare a library of the ancient DNA fragments ligated at both ends to specific DNA adapters for PCR amplification. After isolating the DNA from an ancient specimen, double strand DNA is polished at the 3’ and 5’ ends and is converted in blunt end DNA. The polishing step is performed using simultaneously a DNA polymerase and a polynucleotide kinase that catalyzes phosphorylation at the 5’ position. Library preparation proceeds with the ligation of the adapters to the polished and phosphorylated fragment ends. Adapters consist of short oligonucleotides of known sequence that will permit the design of complementary primers for library amplification and sequencing.

#### Library amplification

454/Roche and Illumina platforms use different amplification procedures, which are strictly related to their sequencing chemistry. In the case of the 454/Roche platform, the library is amplified in a water-in-oil emulsion PCR (emPCR). Each single DNA molecule of the library is bound to a bead that acts as PCR support, and amplified within an aqueous droplet. In the Illumina system, amplification is carried out by an isothermal bridge amplification process run on a glass slide, termed “flowcell” where molecules are amplified independently, thus generating spatially distinct clones, named “clusters”. While in the Illumina system, the same support i.e. the flowcell is used for both amplification and sequencing, in the 454/Roche system, these two steps are performed on different supports. Further details about amplification procedures are reported in Additional file 3 [see Additional file 3. Precise library quantification is a critical step for both platforms, to obtain high quality sequences [105]. For the Illumina system, an appropriate number of clusters must be generated during library amplification, while for the 454/Roche system only a precisely quantified library will ensure a correct calibration of DNA/beads proportions that fosters the binding of a single DNA molecule per bead and the production of an “in-range” emulsion PCR yield.

#### Sequencing chemistry and throughput

The 454/Roche and Illumina platforms use protocols based on different chemistries and produce different numbers of sequenced bases per run. The strategy to generate reads in the 454/Roche system relies on pyrosequencing, based on a single nucleotide addition (SNA) technique [106,107]. Pyrosequencing consists in an enzymatic cascade that is activated each time a nucleotide is added to the growing DNA chain. Each nucleotide addition generates a spot light that is measured by a CCD camera. The intensity of light emitted by the enzymatic cascade is proportional to the number of added nucleotides and is digitalized and converted in nucleotide sequence. Pyrosequencing reactions take place on a fiber glass support, named “PicoTiterPlate” (PTP), on which DNA beads are loaded after emPCR. The updated version of the 454/Roche GS-FLX platform, Titanium Plus, reaches a read length of 800 bases and each run can generate up to 1 Gigabase of sequence. Conversely, the Illumina platform exploits a sequencing-by-synthesis (SBS) strategy, in which modified nucleotides, called cyclic reversible terminators (CRT), are inserted during the synthesis reaction. The different fluorescence emissions by each dye-labeled nucleotide, after laser excitation, are detected by a CCD camera and thus each cluster on the flowcell is associated to a raw sequence. The Illumina GAIIx platform has a sequencing yield of about 50 Gigabases per run, while the updated version HiSeq reaches 200 Gigabases per run. The Illumina platform provides sequence reads with a length ranging from 36 to about 150 bases.

#### Sequencing approaches

In a study on ancient DNA, different types of DNA target can be sequenced depending on the project goal and the available ancient material.

##### Shotgun sequencing

A shotgun sequencing project is performed when isolated DNA is sequenced without any *a priori* selection. The shotgun approach has the capacity of identifying all the known species when total DNA is isolated from bone, teeth or shaft specimen. Given the nature of these sample types, the amount of endogenous target DNA can be very low, because of bacterial or fungal contamination. The shotgun approach is also used for metagenomic studies, when the goal of the sequencing project is to identify all possible known organisms present in an isolated specimen. This approach has been applied by Poinar [108] and Miller [109] to sequence mammoth DNA and they have confirmed the presence of a large amount of exogenous DNA. All the sequences obtained in a shotgun sequencing project are usually identified by matching to sequence databases, with a *“blasting”* procedure, that uses *BLAST*. Due to the high statistical power needed to specifically recognize the reads obtained, the shotgun approach requires a high level of sequencing depth, e.g. the total number of bases sequenced has to exceed largely the total length of the target DNA so that each DNA region is sequenced many times. Matching parameters must be carefully chosen to avoid non specific results, as described in the “Data analysis” section.

Considering the cost of this approach, the currently elected sequencing platform is the Illumina Genome Analyzer system, which can generate millions of reads at a lower cost per base compared to the 454/Roche sequencer.

Due to the high level of bacterial contamination in the shotgun approach, a strategy to decrease the amount of microbial DNA sequences has been proposed by Green et al. [11]. It consists in an enzymatic digestion of the sequencing library with a mixture of restriction enzymes capable of degrading DNA fragments with a GC composition similar to that found in bacterial genomes. Green et al. applied this treatment before library amplification and increased by about 5-fold the amount of Neanderthal DNA in sequencing libraries prepared from archeological remains. By applying this method, the same researchers have recently obtained the draft of the entire genome of two extinct hominin groups: Neanderthals and Denisovans [11,110].

##### Amplicon sequencing

PCR is the most efficient procedure to selectively select a target DNA region. PCR includes a series of well-known steps that need to be optimized for each specific application. The most important step is designing primers, which must take in account the specificity needed to obtain robust results. PCR advantages and disadvantages must be considered before deciding on the rationale of an ancient DNA sequencing project. The most important advantage of a PCR approach is that, via DNA amplification, a high amount of material is obtained i.e. PCR products or amplicons, which are easily converted in an NGS library that can be sequenced. Even if the specificity of PCR is very high, a major disadvantage is the increased rate of misincorporations in the fragment ends, which are important markers for ancient DNA studies, as discussed later in this review.

PCR should be used when the sequencing target is well known and the goal is to detect SNP or small variants used as markers for haplotyping, for instance to identify Neanderthal private nucleotide substitutions. This strategy has been used to identify nucleotide variants in short sequences of specific genes like those determining blood group, taste perception and brain development in Neanderthal samples [111-113]. In a multiplex PCR strategy, different amplicons can be mixed together in a balanced mixture and sequenced at the same time. A multiplex PCR strategy, coupled with NGS sequencing, has made it possible to characterize the entire mitochondrial genome of the Tyrolean Iceman Ötzi, one of the best conserved mummies [114], and to identify the mitochondrial haplotype, K1ö, observed for the first time in this specimen. Another study comparing this sequence with modern human PCR products characterized precisely the nucleotide misincorporations in the mitochondrial DNA of this Tyrolean Iceman Ötzi [115]. Similarly, a PCR multiplexing strategy was used to recover the complete mitochondrial genome sequence of extinct animals, such as cave bears [116] and Auroch (*Bos primigenius*), an ancestor of domestic cattle [117].

##### Sequence capture

Sequence capture is a methodology that uses specifically designed probes to recognize and capture a target DNA, permitting both sample enrichment and recovery of information on DNA misincorporations at the 3’ and 5’ fragment ends.

**Figure 3:**
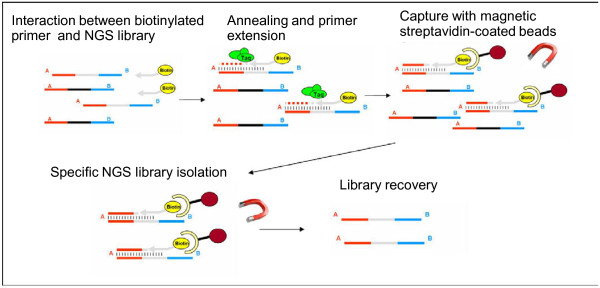
**Primer extension capture.** Specific genomic regions are targeted by hybridization between biotinylated capture primers and NGS library sequences; the strand extension and the capture step allow the recovery of the enriched library.

The first capture methodology used in the field of ancient DNA was the primer extension capture (PEC) approach, which uses biotinylated primers specifically designed to identify particular regions and to permit extension until the end of the DNA fragments [118] (Figure 3). Such primers have been exploited by Briggs et al. [118], to capture and sequence the entire Neanderthal mitochondrial genome from whole DNA isolated from an ancient specimen and converted into a labeled NGS library. Once the biotinylated primers bind to the specific target regions contained in the library (e.g. target species mtDNA), an extension step is performed using a DNA polymerase that synthesizes the complementary strand until the end of the DNA fragment. This extension step increases the specificity of the biotinylated primers. The specifically annealed fragments are captured by streptavidin-coated beads that bind the biotinylated-primer/target-library-fragment complexes. Then, a melting step recovers the captured fraction of the initial library, which represents a small but enriched fraction of the whole DNA isolated from the specimen. This library is finally amplified and sequenced with NGS.

This procedure has a very high specificity and has in fact, improved the capacity to recover complete mtDNA from very complex samples such as Neanderthal and other hominin bone fragments [13,118,119], but synthesizing biotinylated primers for an entire mtDNA sequence is still an expensive step. To decrease the cost of the PEC approach, it is possible to either target only a few short regions or to substitute primer synthesis for probes generated by a cheaper procedure. As shown by Maricic et al. [120], PEC probes can be generated by fragmenting and then biotinylating long products obtained by the amplification of modern mtDNA. This approach makes it possible to obtain PEC probes starting from any target region, whether mitochondrial or nuclear DNA, isolated from human or other animal samples.

Another capture approach uses biotinylated probes longer than a standard primer, and thus an extension step is not needed and information on misincorporations at the ends of the fragments can be enriched and recovered.

**Figure 4:**
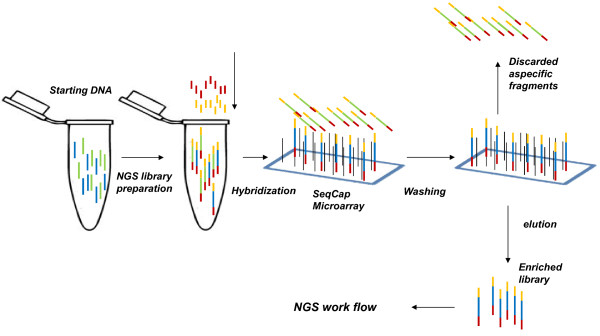
**Nimblegen sequence capture workflow.** In solid-sequence, capture is performed by hybridization between NGS library sequences and the capture probes immobilized onto an array surface.

**Figure 5:**
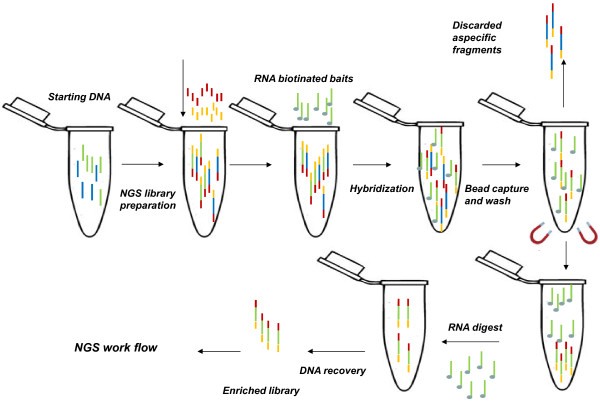
**Agilent SureSelect sequence capture workflow.** In solid-sequence, capture is performed by hybridization between NGS library sequences and the capture probes immobilized onto an array surface.

Probe enrichment methodologies are already available for modern human, mouse, dog and cattle DNA re-sequencing projects, which are applied either to study specific genomic regions genetically related to diseases or to analyze a whole exome (i.e. coding exonic DNA) [121,122]. These capture methodologies are available in two forms, either in solid- or in liquid-phase. The solid-phase approach relies on probes immobilized onto an array surface [123], like those used in microarray gene expression studies. Nimblegen (SeqCap arrays) and Agilent (Agilent 244 K microarrays) have developed this kind of approach (Figures 4 and 5 respectively). Burbano and coworkers [124] have used a Nimblegen modified array version for the analysis of ancient DNA. This procedure uses a microarray surface on which probes specifically designed to capture specific genomic regions are immobilized. The NGS library is then hybridized onto the array surface so that the probes capture the targeted DNA regions. After hybridization, non-specific DNA fragments are washed away, while the library DNA fragments complementary to the probes are subsequently recovered by an elution step. These fragments represent a small and selected fraction of the entire genome. The library enrichment ratio depends on the sizes of the captured genomic regions and of the entire genome. Nimblegen has also developed a commercial capture array, which can capture the whole human exome, with 2.1.10^6^ immobilized probes, for a total of 30.10^6^ captured bases. Considering the human genome size, this capture array allows a ~100-fold enrichment.

The Agilent in liquid-phase approach i.e. SureSelect target enrichment system, uses biotinylated RNA probes, named “baits” that are subsequently captured by streptavidin-coated beads [125]. This procedure is similar to that of Nimblegen, but the supporting material for capture consists of beads suspended in a buffer, instead of a solid array. The in-liquid selection has a higher capture yield due to a better interaction between the library and the probes. Another in liquid-phase approach developed by CustomArray uses biotinylated probes synthesized in a customized way using *in situ* synthesis. The DNA probes are then converted into RNA probes and biotinylated to permit enrichment of the target DNA and collection. Finally, the captured enriched library is quantified and sequenced by NGS.

### Data analysis

#### Reference sequence: de-novo or re-sequencing

In general, sequencing projects can be separated into “de-novo” and “re-sequencing” projects, depending on the nature of the sample analyzed and the availability of a reference sequence. In fact, since a reference sequence is required to assemble short reads of damaged DNA, ancient DNA sequencing projects are re-sequencing projects. When a new ancient species is investigated it is necessary to decide which sequence(s) to use as reference. For anthropological studies, the *Homo sapiens* genome reference sequence is used even when the sequenced ancient DNA originates from a close relative, such as Neanderthal [11] or Denisovan [119]. In these cases, a double mapping onto the human and a close primate genome reference sequences may be required to identify the correct position of the hominin sample in the evolutionary tree. Studies on wild ancestors of modern species generally use the reference sequence of the corresponding modern species [108,117,126].

The large number of on-going sequencing projects and the availability of sequences in public databases, make re-sequencing a widespread approach. However, de-novo sequencing differs from re-sequencing because it requires the generation of an informative and robust sequence with a higher sequencing depth. Thus, the generation of different libraries with different fragment sizes and the assembly of the reads involve the use of specific *ad-hoc* algorithms that can reconstruct a previously unknown sequence.

##### Mapping of reads

Re-sequenced DNA can be analyzed to identify nucleotide variations specific to a given species or to discriminate between phylogenetically-related species. For example, Neanderthal private polymorphisms in the HVR-1 mitochondrial region are used as markers to discriminate between *Homo sapiens* and Neanderthal DNA [13]. Sequence variations are detected by mapping reads from ancient DNA on a reference sequence using BLAST. Any nucleotide difference between the reference sequence and the ancient DNA reads is a putative private variation. Two important BLAST parameters must be considered to obtain robust results: the gap opening penalty (*g*) and the e-value (*e*). When analyzing ancient DNA sequences, it is necessary to avoid setting too stringent matching parameters, to increase the probability of having an informative result. Moreover, sequencing errors must be taken into account when choosing matching parameters. Illumina and 454/Roche have different patterns of sequencing errors. On the one hand, Illumina sequencing is associated with a high error in base calling, which leads to a high rate of false base substitutions. These errors can be detected and corrected by sequencing at a sufficient depth (20X or more). On the other hand, the 454/Roche pyrosequencing reaction cascade accumulates insertion or deletions (INDELs) within homopolymeric regions resulting in a sequence of low quality in these regions. Thus, putative variants detected in these regions will require further validation. Given the sequencing error rates of these two NGS platforms, many sequencing projects combine the two technologies to compensate for their respective limits.

Considering all the difficulties associated with sequencing ancient DNA, such as nucleotide misincorporations and NGS error rates, matching parameters G and e-values, for reads mapping should be carefully chosen. For these reasons, the parameter G that is related to the space introduced during the alignment between two sequences and represents the cost to open a gap is usually set to low values. Moreover, the e-value that is a parameter related to the reliability of the similarity between two sequences is expected to be very low for specific matches. Matching results must be filtered to discard data with non-specific matches.

##### Mind the gap

The origin of the reads is variable since they can originate from both ancient and modern DNA fragments present in the analyzed sample. Currently, different criteria are used to recognize and discriminate ancient from modern DNA. Ancient DNA fragments are usually short, less than 120 bp long, but this is not sufficient to distinguish between ancient and modern DNA. Another important feature of ancient DNA is the presence of miscoding lesions at the 5’ and 3’ ends of the fragments [127], where a higher frequency of base substitutions from C to T and from G to A is observed compared to that in modern DNA (approximately 35 % in Pleistocene ancient human samples). Thus, a reduced fragment length associated with the occurrence of terminal miscoding lesions is the main feature used to distinguish ancient from modern DNA. If a high sequencing depth is available, DNA damage rate can be calculated with the “mapDamage” software [128]. Contamination from modern DNA requires special attention when hominin specimens are investigated. Even if the sample’s taphonomic history is well known, putative contaminations from modern human DNA can reduce the number of informative reads. The shotgun approach can discriminate between target and contaminant reads, but the cost to obtain robust results can be very high. Using a selection procedure such as sequence capture can reduce the spectrum of target sequences, while keeping information on miscoding lesions in fragments of ancient DNA.

#### Applications of NGS technologies in the field of ancient DNA

##### From the mammoth to the Neanderthal genome project

The first publication on ancient DNA sequencing using an NGS platform was entitled “Metagenomics to paleogenomics: Large-scale sequencing of mammoth DNA”, published by Poinar et al. in *Science* at the end of 2005 [108]. Poinar and colleagues performed a metagenomic study on a mammoth bone sample using the first version of the 454 sequencer and produced 302 692 sequence reads with a mean length of 95 bp, for a total of nearly 30.10^6^ bases. After this milestone publication, many other sequencing projects of ancient DNA have been carried out based on high-throughput next-generation sequencing.

In May 2006, the first nuclear DNA sequences from a Neanderthal (*Homo neanderthalensis*) were reported by Pääbo [129], as part of the Neanderthal Genome project that had started about two years before. Within this project, Pääbo and coworkers probed 60 Neanderthal specimens from museums to investigate the degree of DNA preservation after thousands of years. Two of the specimens analyzed provided interesting results and Pääbo’s team reported at the Biology of Genomes meeting at New York's Cold Spring Harbor Laboratory that they had succeeded in sequencing about 10^6^ bpof nuclear DNA (about 0.03 % of the genome) from a 45 000-year-old male from the Vindija cave, Croatia using pyrosequencing. Another report by James Noonan at the Cold Spring Harbor meeting in the same year, presented a preliminary analysis of 75 000 of the 10^6^ bp sequenced so far. These two papers [129,130] on Neanderthal nuclear DNA sequences from fossil bones promise to answer questions on the relationship between extinct species, such as Neanderthals and present-day humans. Typically, one of these questions is whether the Neanderthals are the direct ancestors of modern-day humans or an evolutionary side branch that eventually died out. Although these two studies were the first to investigate *Homo* ancient DNA on a large scale, they came to very different conclusions regarding the ancestral role of Neanderthals. A year later, Wall and Kim [131] reanalyzed the data from these two original studies and found that their results contradict each other. This implies that the data from at least one of the studies are probably incorrect, due to contamination by modern human DNA, which strongly compromises the final findings [131].

Recently, Green et al. [11] sequenced a large part of the Neanderthal nuclear genome using NGS. Starting from about 400 mg of bone powder, they generated 5.3 Gigabases of Neanderthal DNA sequences with a 1.3X genome coverage. DNA sequences matching at a significantly higher rate to primate genomes than to any other organism were further analyzed. These results revealed that Neanderthals and present-day humans shared a common ancestor about 800 000 years ago, which diverged between 270 000 and 440 000 years ago. This study also showed that Neanderthal DNA shares more genetic variants with present-day humans from Eurasia than from sub-Saharan Africa i.e. on average 2.5 % (range 1–4 %) of the genome of people outside Africa derive from Neanderthals. In addition, the authors provided a list of genomic regions and candidate genes that could have been under early positive selection pressure in modern human history, for example, those involved in cognitive abilities and cranial morphology.

##### Denisova man

Along these same lines, another important study has shed new light on the evolutionary history of the *Homo* genus. In 2010, Krause et al. have described the distal manual phalanx of a juvenile hominin excavated from the Denisova cave (Altai Mountains, southern Siberia) [119]. The phalanx was found in layer 11, which has been dated 50 000 to 30 000 years ago. This layer contained micro blades and body ornaments of polished stone, typical of the ‘Upper Palaeolithic industry’ and generally thought to be associated with modern humans, and also stone tools that are more characteristic of the earlier Middle Paleolithic age, such as side scrapers and Levallois blanks. A sequence capture approach [118,119] combined with high-throughput sequencing [104,119] was used to characterize the complete mtDNA genome from this specimen.

To clarify the relationship between the Denisova individual and other hominin groups, Reich et al. have sequenced the nuclear genome of the Denisova specimen and analyzed its genomic relationship with Neanderthal and present-day human genomes [110]. Two independent sequencing libraries were generated from the ancient DNA extracted from this specimen, using a modified Illumina protocol. The results show that the Denisova individual belongs to a hominin group, named the “Denisovans”, which share a more recent common ancestor with Neanderthal but have a population history distinct from both Neanderthals and modern humans. The major outcome of this study is that this is the first time that a new population, the “Denisovans” is characterized starting from DNA. In fact, at present, except for the phalanx and a tooth retrieved during the archaeological excavation, no other physical and morphological feature of this population is known. Additional important issues have been addressed in a study on archaic hominins taking into consideration the important argument of gene flow between modern and extinct humans, as reported by Lalueza-Fox et al. [132].

##### Modern human evolution

With the advent of the NGS technology and the possibility to reconstruct the genome of extinct hominins, new perspectives to study human evolution have opened up. Malmström et al*.* have studied the dynamics of a Scandinavia population during the Neolithic era by sequencing DNA from ancient Scandinavian human remains [133]. Gilbert et al. have sequenced the mtDNA of a Paleo-Eskimo human starting from a 3400 to 4500-year-old frozen hair, thus documenting human expansion into the New World's northern extremes [134]. The same DNA source (i.e. hair shaft) had already been used by Gilbert et al. [135] to sequence the mitochondrial genome of a Siberian mammoth (*Mammuthus primigenius*) using NGS. Interesting results have been reported by Rasmussen et al. who sequenced almost 80 % of the genome of a 4000-year-old human genome from Greenland [136]. Besides from showing that the Saqqaq population had no or little European contribution in their genome, this sequencing project identified specific phenotypic traits starting from genomic information, providing important information about an extinct culture. Although these genomic data are obtained from a single individual, a demographic history could be reconstructed.

##### Domesticated and extinct species

Using NGS, it is possible to detect traces of the intermingled genetic histories of the humans involved in the Neolithic processes that led to the domestication and diffusion of a species, as shown by the sequencing of the mitochondrial genome of *Bos primigenius*[61,117].

Using the Roche/454 platform, Ramírez et al. [26] have sequenced DNA isolated from a 6000-year-old bone sample from an extinct caprine species (*Myotragus balearicus*) from the Balearic Islands and thus shown that NGS applied to DNA from extinct animals can lead to a better understanding of adaptation.

Other extinct animals, such as the New Zealand moa have been characterized by Allentoft et al. [137]. They have demonstrated the feasibility of studying ancient biodiversity and extinction processes using NGS. In addition, Miller et al. [138] have analyzed the Tasmanian tiger (*Thylacinus cynocephalus*), extinct since 1936, by sequencing DNA isolated from a museum specimen collection.

#### Third Generation Sequencers (TGS) and ancient DNA: from the past to the future

With the development of new sequencing chemistries and innovative signal detection methodologies, a new generation of sequencers, named Third Generation Sequencers (TGS) have recently become available (Table 2). These novel platforms differ in sample preparations and sequencing chemistries, but all share the particular and innovative feature of direct sequencing, allowing single molecule analysis, hence bypassing PCR amplification. For studies on ancient DNA, the possibility of using very small amounts of starting DNA field is highly appreciated, since it improves detection of DNA damage and identification of contaminants and exogenous sequences. Recently, Orlando and colleagues [139] have applied this new technology to perform single-molecule sequencing of DNA isolated from a Pleistocene horse bone. They used the total DNA isolated from the specimen, without any kind of sequence enrichment (i.e. sequence capture), or PCR amplification, thus allowing an assessment of postmortem DNA damage. In spite of a very high number of sequenced bases, the level of sequence depth was too low (1X on the mitochondrial genome) to proceed further with the analysis Table 2.

**Table 2:** Third Generation Sequencers (TGS): an overview

**Company name**	**TGS sequencing chemistry**
Helicos Genetic Analysis Platform	Virtual Terminator nucleotides
Pacific Biosciences	Anchored DNA polymerase + Zero-mode waveguide (ZMW)
VisiGen Biotechnologies	Modified DNA polymerase + Fluorescence Resonance Energy Transfer (FRET)

Company name and corresponding technology are listed

## Conclusions

The second generation sequencers, such as Roche/454 and Illumina presented in this review but also SOLiD have opened new possibilities for ancient DNA sequencing by providing a very high throughput, which could not be reached previously with traditional Sanger-based platforms. In the last few years, these technologies have made it possible to characterize genomes, metagenomes and transcriptomes, thus rapidly increasing sequence information available for different organisms and applications. The three most important NGS technologies have different specific features positioning each one in a given niche market. Briefly, the Roche/454 technology provides long sequence reads, today up to 800 bases, thus greatly simplifying the genome assembly. The Illumina and SOLiD technologies, thanks to their high throughput, have drastically reduced the cost per sequenced base even if the reads produced do not exceed 100 bases. Although, to date, the SOLiD technology has not been used in studies on ancient DNA, it offers an important asset i.e. high accuracy in base calling due to its two-base encoding sequencing strategy. The throughput of the SOLiD platform, based on a sequencing by ligation (SBL) chemistry is comparable to that obtained by Illumina, but because a reference sequence is required to translate its so-called “color space” into a nucleotide sequence, its application in the field of ancient DNA has not been considered up till now.

The high sensitivity and resolution power offered by the NGS technologies have considerably increased available data on ancient DNA thus greatly improving our knowledge on many interesting issues of human evolution: the history of extinct species, adaptation and domestication processes. New hypotheses and theories have been developed with the most important achievement being the robustness and reliability of sequencing data. Moreover, identification, elimination and estimation of contaminant sequences in ancient DNA samples are issues that need to be discussed and resolved. Developing new molecular biology procedures and innovative *ad-hoc* bioinformatics approaches must be accounted for when planning a sequencing project on ancient DNA. Each feature of an ancient sample needs to be carefully evaluated to develop a specifically focused experimental design in order to optimize the sequencing power of either the NGS or TGS technology used. For this reason, when NGS is used to sequence ancient DNA, we propose to include an additional “golden criteria” to the nine summarized by Cooper and Poinar in 2000 [10]. It concerns the labeling of the ancient DNA sequencing library using synthetic commercial or custom-made nucleotides “TAG”. These “labels” could be project- or sample-specific in the case of shotgun or capture sequencing, to enable estimation of putative contamination of the sample after DNA isolation.

Currently, enrichment protocols of DNA samples and specific bioinformatics pipelines have been successfully developed to increase data reliability. However, other steps in the procedures and analyses need to be optimized to generate a robust ancient DNA data set. NGS technologies and the development of new molecular biology strategies, such as PEC or capture “baits”, have also accelerated the change in the focus of research from the frequently investigated mtDNA (reviewed by Ho and Gilbert [140]) to the more informative nuclear DNA. Major insights have been gained from the multidisciplinary nature of the projects on ancient DNA, which exploit data combined from different technologies capable of generating genome-wide data [141]. Moreover, all the data, which have been used to study complex processes such as human evolution, domestication or demographic events, need to be confirmed by the analysis of many more samples, even if the availability of ancient well-preserved specimens is always a critical and intrinsic feature of palaeogenomic projects.

## Competing interests

The authors declare that they have no competing interest.

## Authors' contributions

DC conceived the project. ER, ML, EG, GDB and DC wrote the manuscript. All authors read and approved the final manuscript.

## Supplementary Material

Additional file 1: **The « Golden criteria » by Cooper and Poinar 2000 [**[10]**].** Click here for file

Additional file 2: How samples should be collected. Click here for file

Additional file 3: **NGS library preparation and sequencing chemistries.** The material provided describes library preparation for the 454/Roche and Illumina platforms and their sequencing chemistries. Click here for file
