# Correction: Ancient DNA studies: new perspectives on old samples

**DOI:** 10.1186/1297-9686-45-4

**Published:** 2013-02-18

**Authors:** Ermanno Rizzi, Martina Lari, Elena Gigli, Gianluca De Bellis, David Caramelli

**Affiliations:** 1 Institute for Biomedical Technologies, National Research Council, Via F.lli Cervi 93, Segrate, Milan 20090, Italy; 2 Department of Evolutionary Biology, Laboratory of Anthropology, Molecular Anthropology/Paleogenetics Unit, University of Florence, Via del Proconsolo 12, Florence 50122, Italy

## Correction

Since the publication of our article (Rizzi et al, Genet Sel Evol, 2012, 44:21), it has come to our attention that our text inadvertently includes some statements taken from the introductory section of the following paper by Pääbo et al. without citation:

Pääbo S, Poinar H, Serre D, Jaenicke-Despres V, Hebler J, Rohland N, Kuch M, Krause J, Vigilant L, Hofreiter M: Genetic analyses from ancient DNA. *Annu Rev Genet* 2004, **38**:645–679.

We apologise for this oversight and any inconvenience this may have caused.
