# Supplementary material for: Ancient DNA studies: new perspectives on old samples
Source: Genet Sel Evol. 2012 Jul 6;44(1):21. doi: 10.1186/1297-9686-44-21 (PMC3390907; doi:10.1186/1297-9686-44-21)
Supplement: Additional file 1: — The « Golden criteria » by Cooper and Poinar 2000 [[10]]. [file 1297-9686-44-21-S1.docx]

**Additional file 1 – The « Golden criteria » by Cooper and Poinar 2000 [10]**

| 1 | Amplification products should be routinely cloned and multiple clones sequenced to unambiguously detect heterogeneity in amplification products and estimate the spectrum of errors. |
| --- | --- |
| 2 | **Blank extractions and several negative PCR controls** should be performed alongside extractions and amplifications from ancient material. In fact, the quantity of DNA contamination present in laboratory reagents may be so small that it is detected only sporadically in negative controls. |
| 3 | **Repeated amplifications** from the same or different extracts from the same specimen are necessary to detect contamination of a particular extraction or amplification and to identify nucleotide mis-incorporations leading to substantial changes. |
| 4 | **Quantifying the number of amplifiable DNA molecules** present in an extract is necessary to evaluate, if their concentration is so low that during the initial PCR reactions, substantial changes may occur. A number of starting template molecules > 1000 may serve as a rule of thumb. |
| 5 | **An inverse correlation between amplification efficiency and length of amplification** is a very simple indicator of the extent of degradation and blocking lesions present in ancient DNA templates. In general, when the amplification rate of shorter *vs*. longer fragments is the same in ancient and modern samples, then it is likely that the ancient sample is contaminated by modern DNA. Moreover the sequences generated from ancient DNA should make phylogenetic sense i.e. they cannot be a combination of different sequences, resulting from contamination of the samples by exogenous DNA. |
| 6 | Biochemical assays of macromolecular preservation support the claim that if a specimen is sufficiently well conserved, the ancient DNA present in the specimen is preserved. The most widely used assay is the **analysis of the amino-acids present in the specimen**. Samples that contain very few amino-acids, or an amino-acid composition indicating that microorganisms have replaced the original macromolecules, or extensively racemized amino-acids, are unlikely to contain endogenous DNA. |
| 7 | DNA sequences derived from the mitochondrial genome are often present in the nuclear genome. Because **mitochondrial DNA is the molecule of interest** in most projects on ancient DNA, erroneous conclusions can be drawn if a combination of nuclear and mitochondrial sequences is analyzed. To prevent this problem, different primer sets should be used to amplify the same mitochondrial sequences, since it is very unlikely that two different primer sets will preferentially amplify the same given nuclear insertion. |
| 8 | **Replication in a second laboratory** is an additional precaution to exclude the occurrence of a laboratory contaminant that fails to appear in blank extracts and negative PCR controls. In such cases, it is preferable to send samples from a museum or an excavation site directly to the two laboratories independently, so that a potential laboratory contaminant cannot be transferred between the laboratories. |
| 9 | When dealing with human remains, for which contamination is more problematic, **faunal remains can also make good negative controls** for human PCR amplifications i.e. evidence that similar DNA targets survive in associated faunal material is evidence of modern contamination. |
