# Supplementary material for: Ancient DNA studies: new perspectives on old samples
Source: Genet Sel Evol. 2012 Jul 6;44(1):21. doi: 10.1186/1297-9686-44-21 (PMC3390907; doi:10.1186/1297-9686-44-21)
Supplement: Additional file 2: — How samples should be collected. [file 1297-9686-44-21-S2.docx]

**Additional file 2 - How samples should be collected**

| 1 | Whenever possible, samples should be collected at the original (archaeological) site using disposable gloves, facemask, head-dress gown and lab coat. Disposable gloves should be changed between different samples. All the instruments used for collection should be sterile. These practices minimize the risk of contamination by modern DNA and enable immediate and appropriate storage of the samples. |
| --- | --- |
| 2 | **Sampled material should be stored as cool and dry as possible,** preferably at - 20°C. If samples are taken from museum collections, where they have generally been stored at approximately room temperature, they should be in any case stored frozen, since longer periods at room temperature can cause further DNA damage. |
| 3 | If electric or mechanical saws, forceps or scalpels are necessary for sampling, it is important to ensure **proper cleaning of the blades between the sampling of two different individuals.** To prevent cross-contaminations, tools should be sequentially washed with concentrated soap or bleach, distilled water and finally absolute ethanol. |
| 4 | **If contamination of the samples by members of the excavation team, museum personnel** or any other person who have handled the material cannot be excluded, it is recommended to take a saliva sample from each person as a control, to enable genotyping and reciprocal comparison. This step is particularly important when a single sample is investigated. When saliva (or any other tissue) of a suspected potential contaminator cannot be obtained, then other material known to have been in contact with the person should be sampled and analyzed. This important control sample should not be submitted to the decontamination procedures that are carried out for the ancient sample. |
