# Supplementary material for: Ancient DNA studies: new perspectives on old samples
Source: Genet Sel Evol. 2012 Jul 6;44(1):21. doi: 10.1186/1297-9686-44-21 (PMC3390907; doi:10.1186/1297-9686-44-21)
Supplement: Additional file 3: — NGS library preparation and sequencing chemistries. The material provided describes library preparation for the 454/Roche and Illumina platforms and their sequencing chemistries. [file 1297-9686-44-21-S3.doc]

**Additional file 3**

**NGS library preparation and sequencing chemistries**

**Library preparation**

***454/Roche* *procedure***

Since most DNA fragments extracted from ancient remains have a short length, the first step in preparing an ancient DNA library is to create blunt-ended DNA fragments using T4 DNA polymerase and Klenow enzyme and to phosphorylate the 5' ends with T4 polynucleotide kinase. Then, specific double-strand DNA adapters A and B, complementary to the primers used in the subsequent phases of emulsion PCR (emPCR) and pyrosequencing, are ligated on each end of the repaired DNA fragments. Adapter B positioned on the 5’ end carries a biotin label necessary for the selection step. Adapter ligation is a random process and produces three types of DNA fragment-adapter combinations: A-fragment-A, B-fragment-A, (or A-fragment-B) and B-fragment-B. A-fragment-A and B-fragment-B combinations are eliminated in two steps (Figure 1): (1) streptavidin-coated beads are added to capture all ligation products carrying a biotinylated B adapter and the A-fragment-A combinations, which remain in the liquid phase, are discarded; (2) after washing, beads carrying B-fragment-B and B-fragment-A combinations are resuspended in a diluted solution of NaOH that denatures double-stranded DNA; since the B-fragment-B combinations are biotinylated at each end of both DNA strands, they remain bound to the streptavidin-coated beads, whereas for the B-fragment-A (or A-fragment-B) combinations, only the biotinylated strands remain linked to the beads and the non-biotinylated strands are released in the solution; thus, all B-fragment-B combinations can be discarded by removing the streptavidin-coated beads and the remaining solution, containing only single-stranded B-fragment-A DNA can be used for emulsion PCR amplification and pyrosequencing. A modification of this protocol has been proposed to increase the efficiency d of the single-stranded DNA fragments release during the denaturation step [116]. It consists in an additional heat treatment that breaks the links between biotin and streptavidin allowing the recovery of the biotinylated strands that are usually discarded and thus the number of fragments in the library is increased by several folds. Heat-treated libraries will contain biotinylated B-fragment-A strands as well as B-fragment-B and non-biotinylated A-fragment-B single-stranded DNA. A higher number of B-fragment-A combinations is recovered, which improves exhaustiveness of the DNA molecules sequenced from the original small number in the sample, while the B-fragment-B combinations do not impede sequencing since they are not amplified in the emulsion PCR. In addition, by using both NaoH and heat treatments, the sequences of both strands of individual template DNA molecules can be determined, which permits to detect problems of DNA integrity or sequencing errors. In fact, a difference between sample and reference sequence observed on only one strand suggests that either a modification occurred only on one of the two nucleotides that make up the base pairs or a sequencing error occurred on one of the two strands.

Before emulsion PCR amplification, the single-stranded DNA library needs to be accurately quantified.

***Illumina procedure***

In the Illumina procedure, the first step consisting in creating blunt ends and their phosphorylation is identical to that previously described. This is followed by an “A-tailing” adenylation step in which a single nucleotide A is added at the 3’ end of the DNA fragments. Specific “T-tailed” adapters are then ligated to the DNA fragments. The A-tailing reaction and the use of the 3’ T-tailed adapters prevent self ligation of the DNA fragments and adapters during the ligation step. The adapter-ligated library is amplified by a few PCR cycles and then purified to remove unligated adapters and quantified.

***Multiplexing***

For multiplexing, the DNA of each sample is tagged using a unique short nucleotide sequence named “MID” for 454/Roche and “index” for Illumina. In both cases, “MIDs” or “indexes” are ligated during library preparation after the end-blunting step. The level of multiplexing depends on the number of samples to be sequenced and the availability of different tags. At present, both 454/Roche and Illumina provide about 10 to 12 different commercially available tags and in the near future, this number is expected to increase up to about 100 for 454/Roche. Indexes or MIDs ligated to the library’s DNA fragments act as barcodes to distinguish each sample and to detect external contaminants from sample DNA sequences. A project-specific barcoding can be designed, to increase the control of contaminants originating from non ancient material or from other samples treated in parallel, during library preparation.

***Library quantitation***

For both 454/Roche and Illumina technologies, library quantitation is a critical step. The quantitation methods recommended by 454/Roche, such as the Agilent Bioanalyzer mRNA Pico assay or PicoGreen fluorometry have a low reproducibility and low specificity, which has led to suggest alternative methods. As reported by Mayer in 2008 [139]: “…quantitative PCR reduces the material demands of high-throughput sequencing”, so the best quantitation method in terms of reproducibility and specificity, up to now is the quantitative Real Time PCR (qPCR).

**Amplification procedure**

***454/Roche emulsion PCR (emPCR)***

Emulsion PCR is performed on sepharose beads carrying immobilized primers complementary to the B adapters of the library. To obtain a clonal amplification of a single molecule per bead, a 1:1 proportion of library fragments and beads is added to the reaction mixture. Correct library quantitation is crucial in this step since no readable sequence can be obtained from beads carrying either no fragments or more than one fragment.

After capture with the streptavidin-coated beads, a water-in-oil emulsion is created by shaking the mixture of beads, oil and PCR reagents. The water droplets generated by the emulsion act as PCR microreactors and contain biotinylated primers, buffer, nucleotides, salts and the DNA polymerase to amplify each library fragment bound to a bead. During amplification, the immobilized primers are elongated by the DNA polymerase and therefore the PCR products are not released in the solution but remain attached to the bead surface, because library adapters and immobilized primers sequences are complementary. At the end of the emulsion PCR, each droplet contains millions of identical fragments i.e. clones of the single library molecule captured by each bead. The beads are then recovered breaking the water-in-oil emulsion using propanol and ethanol solutions. Due to the stochastic variations occurring during the mix preparation, the emulsion and the PCR reaction, the beads recovered represent a heterogeneous population, consisting of “null beads” i.e. that have captured no library fragments, mixed beads that have captured more than one library fragment and DNA beads that have correctly captured a single library fragment. Null beads and mixed beads will not produce any readable sequence and should therefore be excluded from sequencing. An enrichment step is performed with streptavidin-coated magnetic beads that capture all the beads carrying products having incorporated biotinylated primers during amplification. The null beads are discarded and the positive beads are counted (Figure1). If the number of positive beads is high, and the enrichment yield exceeds the threshold parameter of 20%, the bead population is considered as containing too many mixed beads and discarded. Reactions with an enrichment yield lower than 20% generate DNA beads that are loaded onto a PicoTiterPlate (PTP) and sequenced.

***Illumina bridge amplification***

The Illumina DNA library is hybridized to a solid surface of a glass slide, named “flowcell”, which is the support for both the amplification and sequencing steps (Figure 2). Primers complementary to the library adapters are immobilized on the solid surface and capture the denatured single strand library molecules. Captured strands are extended by DNA polymerase and the double-stranded DNA molecules generated are denatured by an isothermal formamide solution. The neo-synthesized single strands create bridges between their 3’ end sequence and the complementary primers attached to the flowcell surface. Once a bridge is formed, the DNA polymerase generates the complementary strand. Cycles of isothermal denaturation, bridge creation and DNA synthesis generate hundreds of millions spatially separated amplification products, i.e. the “clusters” reported in Figure 2. After cluster generation, the flowcell is loaded into an GA II_x_ instrument for the final sequencing step.

**Sequencing chemistries**

***Pyrosequencing***

The pyrosequencing approach implemented in the 454/Roche sequencing technology is based on the detection of light emission at each single-nucleotide addition (SNA) [140,141]. Since nucleotides are not modified, they are added to the reaction one at a time and non-incorporated nucleotides are discarded before the addition of the next one. In the presence of a complementary base, nucleotide incorporation releases inorganic pyrophosphate (PPi), which is used by the enzyme ATP sulphurylase to transform APS in ATP. ATP is then used by the luciferase enzyme to oxidize luciferin in oxyluciferin, a process that generates a light flash of intensity proportional to the amount of ATP. Apyrase, another component of the enzyme mixture, decreases background noise by converting non incorporated dNTP into dNMP. With the 454/Roche platform, the emitted light is detected and measured by a Charge Coupled Device (CCD) camera that converts the light in a digital signal. As previously mentioned, the light intensity is proportional to the amount of ATP synthesized, which depends on the number of nucleotides added to the growing chain in the same reaction. This permits to sequence short stretches of a same nucleotide (e.g. the addition of three dCTP complementary to a GGG template sequence produces a signal four-fold higher than the addition of a single dCTP).

The DNA beads recovered after emulsion PCR are loaded into the PicoTiterPlate (PTP) i.e. the support for the sequencing reaction [103], which can host about 2 million DNA beads. It is made of fiberglass, to enhance light detection and it has been further improved with the Titanium version, in which the inner side of the PTP wells is covered by a thin layer of titanium. Titanium has the property of increasing the focus of the light emitted from the wells hosting DNA beads.

Recently, 454/Roche has upgraded its pyrosequencing chemistry efficiency, reaching a read length of 400 bases in the Titanium version, and 800 bases in the updated version (FLX+) of the platform. Pyrosequencing shows a very low base substitution error rate, but it is quite imprecise in the sequencing of homopolymeric regions exceeding the length of a few nucleotides. The 454/Roche Titanium version has a throughput of about 500 million bases per run while the GS FLX+ version, which generates 800 bases long reads has a throughput of about 1 Gigabase.

***Sequencing by synthesis (SBS)***

The Illumina Genome Analyzer (GAII_x_) uses a sequencing chemistry similar to the standard Sanger dideoxynucleotides methodology. Illumina Sequencing by Synthesis (SBS) exploits cyclic reversible termination (CRT) based on the use of fluorescent reversible 3′ blocked terminators. Unlike the Sanger method, that uses a mixture of unlabeled deoxy- and labeled dideoxy-nucleotides, the CRT method uses only labeled and blocked nucleotides that do not permit further strand elongation. The nucleotide incorporated into the growing chain is identified by fluorescence imaging. In CRT, both the blocking and labeled nucleotides are reversible and can be cleaved to remove the terminating/inhibiting group and the fluorescent dye. The cyclic repetition of blocked nucleotide addition, fluorescence detection and cleavage, permits the identification of the incorporated nucleotides (Figure 2). The number of cycles determines the read length. The updated version of the Illumina sequencer (HiSeq) provides a reads length of 150 bases. The sequencing reaction occurs on flowcells containing clusters generated by bridge amplification. The error rate of the Illumina sequencer is high for base substitution, about 1 per 100 sequenced bases, but this is compensated by a very high throughput since the GA IIx and the HiSeq can produce up to 50 and 200 Gigabases per run, respectively.
